# Efficacy of optimal nutraceutical combination in treating PCOS characteristics: an in-silico assessment

**DOI:** 10.1186/s12902-024-01571-y

**Published:** 2024-03-29

**Authors:** Abha Saxena, Manali Sherkane, Rachana Bhoite, Manasa Premasudha Sadananda, Vinita Satyavrat, Venkatesh Kareenhalli

**Affiliations:** 1 MetFlux Research Pvt Ltd, Mumbai, India; 2 Dr Reddy’s Laboratories Pvt Ltd, Hyderabad, India

**Keywords:** In-silico, Intervention, Nutraceutical, Polycystic ovary syndrome (PCOS), Systems-biology

## Abstract

**Background:**

Polycystic ovary syndrome (PCOS) is a serious health condition affecting women of reproductive age. High prevalence of PCOS and associated metabolic complications needs effective treatment and management. This study evaluated the efficacy of optimal nutraceutical combinations in improving PCOS characteristics using system biology-based mathematical modelling and simulation.

**Methods:**

A shortlisting of eight potent nutraceuticals was carried out with literature search. Menstrual cycle model was used to perform simulations on an in-silico population of 2000 individuals to test individual and combined effects of shortlisted nutraceuticals on five PCOS characteristics [oligomenorrhea, anovulation, hirsutism, infertility, and polycystic ovarian morphology (PCOM)] for a duration of 6 months. Efficacy was tested across lean and obese phenotypes and age groups.

**Results:**

Individual assessment of nutraceuticals revealed seven most potent compounds. Myo-inositol among them was observed to be the most effective in alleviating the PCOS characteristics. The in-silico population analysis showed that the combination of melatonin and ALA along with myo-inositol was efficacious in restoring the hormonal balance across age-groups and Body Mass Index (BMI) categories.

**Conclusion:**

Supplementation with the combination of myo-inositol, melatonin, and ALA demonstrated potential in managing PCOS symptoms in our in-silico analysis of a heterogeneous population, including lean and obese phenotypes across various severities and age groups, over a 6-month period. Future clinical studies are recommended to validate these findings.

**Supplementary Information:**

The online version contains supplementary material available at 10.1186/s12902-024-01571-y.

## Background

With a global prevalence of 1.55 million incident cases, polycystic ovary syndrome (PCOS) has become the most common gynaecologic endocrinopathy [1]. PCOS is characterized by diverse disorders such as hyperandrogenism, polycystic ovarian morphology (PCOM), oligo-anovulation, insulin resistance-led hyperinsulinemia and dyslipidaemia [2]. While the underlying pathological mechanisms are still unclear, it is suggested that insulin resistance (IR) and hyperandrogenaemia (HA) are central to PCOS pathogenesis [3]. Insulin resistance-led hyperinsulinemia primarily causes HA and leads to alteration of steroidogenesis in ovary and in adrenal glands [4]. Moreover, in PCOS, premature responsiveness to luteinizing hormone (LH) arrests follicular development due to altered steroidogenesis [4]. The tendency of granulose cells to secrete anti-Müllerian hormone (AMH), leads to elevated serum concentration of AMH. High circulating AMH desensitizes ovarian follicles to follicle stimulating hormone (FSH), and subsequent premature follicular arrest [5].

PCOS not only lead to impaired fertility but also cause adverse sequelae. Women and adolescent teenagers with PCOS also suffer from metabolic syndrome [6]. PCOS is observed to evolve from a reproductive disease to a more metabolic disorder as the age advances [7]. Abdominal obesity, lipid disorders, diabetes mellitus, hypertensive and cardiovascular disorders, and endometrial cancer may appear as long-term consequences [7]. Therefore, the rising prevalence of PCOS is a concern. Incidence rate of PCOS has increased by 1.45% from 2007 to 2017 globally [1]. Various studies from India have estimated prevalence of PCOS between 3.7 and 22.5% [8] with majority of cases being reported from urban India [9]. As per an observational study conducted by Bahadur et al. (2020), PCOS usually manifests as ovulatory dysfunction, PCOM and HA in Indian reproductive age women [10].

In most cases, PCOS is diagnosed only when clinical complications appear. These complications consist of menstrual irregularities, infertility, hirsutism, acne, and alopecia [2] which altogether reduces the quality of life in patients [11]. Early intervention is hence needed to attenuate adverse outcomes related to PCOS [12].

At present, PCOS management prioritizes on improving clinical symptoms such as ovulation, hirsutism, menstrual irregularity as well as regulating sexual hormones and insulin levels. Currently, metformin and combined oral contraceptives are used as the primary treatment option because of their proven effectiveness in alleviating disease symptoms [13]. However, the consumption of metformin is associated with gastrointestinal side effects, vomiting, and nausea [14]. Besides, longitudinal follow-up studies observe high dropout rates during metformin treatment [15]. Such shortcomings of conventional treatments have led to an increase in invitro and in vivo research of potential nutraceuticals. Nutraceuticals broadly refer to biologically active components typically found in food, but presented in a non-food format (usually as dietary supplements) with the intention of promoting and improving health [16]. They have come across as promising pharmacological agents in improving insulin resistance, disordered lipid profile, inflammation, and related disruptions in molecular pathways involved in PCOS [17].

The present study aims to identify an effective combination of nutraceuticals in PCOS symptom management using system biology-based mathematical modelling and simulation. In-silico quantitative analysis allows a focused approach to identify the potential compounds based on published data. Model-based validation of the effect of these identified nutraceuticals on PCOS severity enable prior impact assessment that can reduce the clinical trial costs. It was hypothesized that the derived optimal combination of nutraceuticals would improve the PCOS symptoms in a model-simulated PCOS population.

## Methods

### Shortlisting of compounds to identify potential nutraceutical combinations

For the purpose of this study, we defined nutraceuticals as vitamins, vitamin-like compounds or polyphenolic compounds having a role in alleviating PCOS symptoms. Eight efficient nutraceutical compounds were identified from the published literature and data analysis (Inositol, vitamin E, coenzyme Q, N-acetyl cysteine, alpha-lipoic acid (ALA), L-carnitine, melatonin and Silybin). These compounds are reported to address most of the known symptoms and physiological perturbations of PCOS. Details of the shortlisting process are mentioned in the supplementary file (Fig. S1). The nutraceuticals were shortlisted by filtering out the compounds which had insufficient data [17–22] or compounds associated with potential side-effects [23–28]. Few compounds were eliminated from investigation due to uncertain efficacy [29–33] and non-reproducible results [23, 34]. The effect of these eight nutraceuticals is assessed on an in-silico PCOS population for their individual and combined effects.

### Computation model description

The model is based on menstrual cycle irregularities in PCOS [35]. It represents dynamics of the hypothalamus-pituitary-ovarian axis (HPO-axis) and distinct stages of ovarian development. The model simulations could predict mass of pre-antral follicle (PrAP) and serum levels of LH, Estradiol (E2), and Testosterone (T) hormones in PCOS condition. In the present study, simulations were performed to capture the nutraceuticals effect on PCOS characteristics when administered individually and in combination. Based on the mechanism of action of nutraceuticals, the associations were made for each model subsystem parameters. Inositol, L-carnitine, and melatonin are observed to improve ovarian function [36–38] and have been associated with ‘k2’, ‘beta’ and ‘m1’ parameters from ovarian subsystems [35]. N-acetyl cysteine and coenzyme Q have a role in glucose metabolism [39, 40] and are associated with parameter ‘cFSHe’ from FSH subsystem [35]. Vitamin E lowers testosterone levels [40] and has been associated with parameter ‘cLHe’ from LH subsystem [35]. Silybin is known to improve the PCOS-related non-alcoholic fatty liver condition [36] and has been associated with ‘epsilon’ parameter from ovarian subsystem [35] and ALA is known to improve lipid profile [41] and associated with rFSH parameter [35]. To obtain the optimal combination and effective dosage of nutraceuticals, the pharmacokinetic profile of each nutraceutical was simulated for six months through associated parameters for different doses using the model to study the physiological effect of nutraceuticals on PCOS characteristics.

### Scoring system of PCOS

Among the five PCOS characteristics i.e., oligomenorrhea, anovulation, hirsutism, infertility and polycystic ovaries, presence of at least 2 characteristics was considered necessary to establish PCOS condition in the model. This criterion is in line with the published literature for PCOS diagnosis [2]. The model considered hormonal peak in ovulatory phase for obtaining LH levels while E2 levels were obtained as mean of ovulatory and luteal phase. The length of the menstrual cycle was obtained from the time period between two consecutive LH surges and was used to score oligomenorrhoea and anovulation [42]. E2 is known to support ovulation and pregnancy [42]. Hence, its levels were used to score infertility. T levels were used to score hirsutism while the mass of PrAP was used to score polycystic ovaries [43].

### In-silico population generation

Heterogeneous in-silico population of 2000 women with PCOS were simulated using delay-differential equation model discussed above. The developed population consists of both lean and obese PCOS phenotype of reproductive age-group (18–40 years). The simulated population was categorized into lean and obese PCOS based on serum triglyceride levels. Populations with triglyceride levels less than 150 mg/dl were considered in lean PCOS (46.06%), whereas those having triglyceride levels greater than or equal to 150 mg/dl were considered in obese (53.94%) PCOS. The thresholds for triglycerides were referred from published studies [44]. PCOS individuals varied in terms of cycle length, preantral follicle count, insulin resistance and levels of LH, T, E2, AMH and homeostasis model assessment-estimated insulin resistance (HOMA-IR).

## Results

### Healthy versus PCOS simulation

**Fig. 1:**
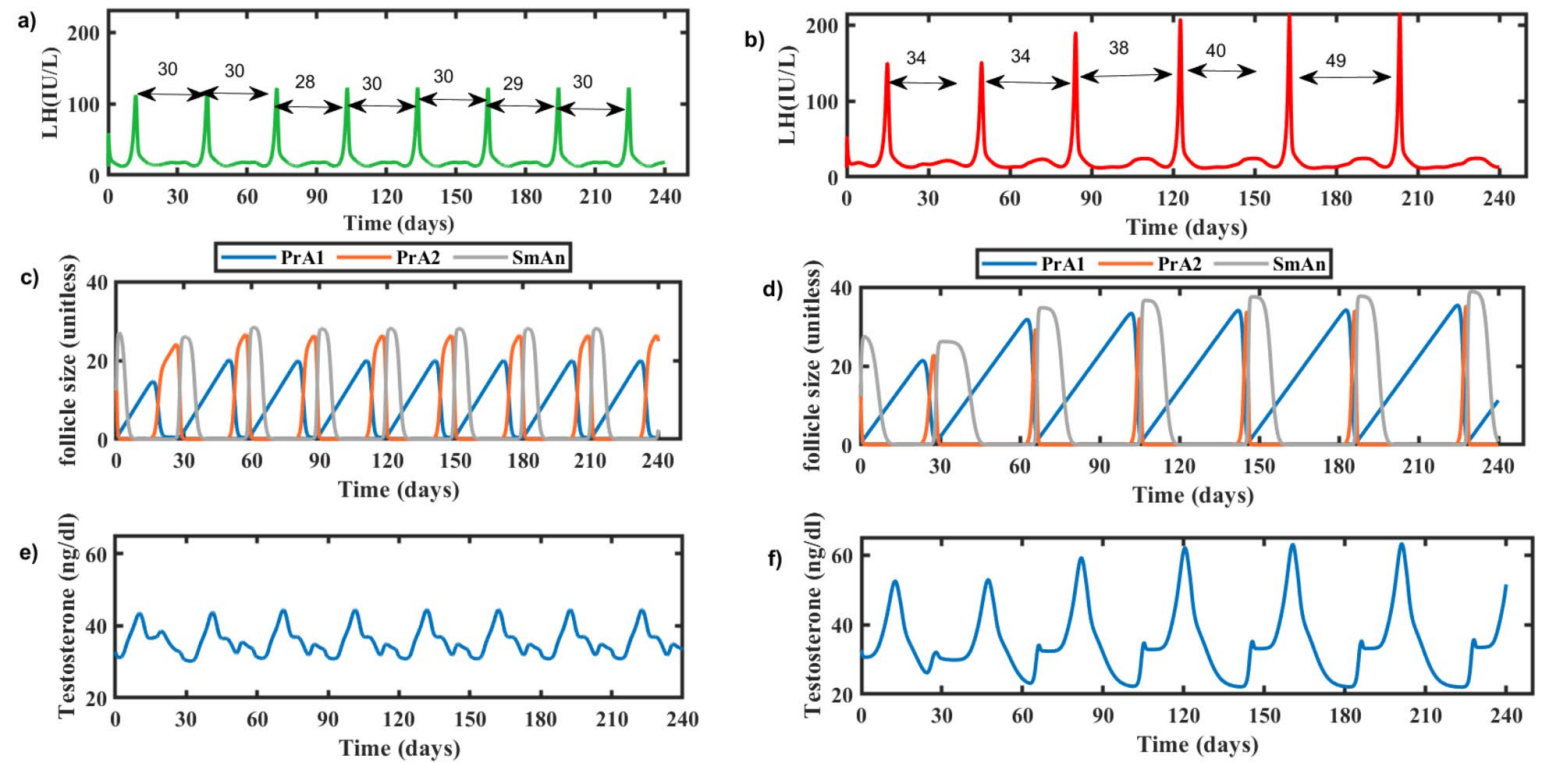
Simulation of healthy and PCOS physiological state for 7 months. (**a**) LH levels in healthy state, (**b**) LH levels in PCOS state, **c**) mass of antral follicles in healthy state, (**d**) mass of antral follicles in PCOS state, (**e**) serum testosterone levels in healthy state, (**f**) serum testosterone levels in PCOS state. PrA1: preantral follicle 1, PrA2: preantral follicle 2, and SmAn: small antral follicle

PCOS state was simulated by perturbing essential pathophysiological parameters. Frequency of LH peaks and the level of variation in two consecutive LH surges were linked to the number of menstrual cycles in 7 months and the length of cycle, respectively (Fig. 1). The seven-month duration shows seven cycles in a healthy state and 6 cycles in PCOS state (Fig. 1 and b). Reduced frequency of cycles indicates an increased cycle length (from 30 days to 35 days) leading to oligomenorrhea in PCOS. LH hormone levels were elevated in case of PCOS state (190 IU/L) in comparison to healthy state (120 IU/L). The mass of the pre-antral follicle increased from healthy to PCOS, resulting in multiple immature follicles (Fig. 1c and d). Also, the testosterone hormone level was high in PCOS compared to the healthy state (Fig. 1e and f).

### Prevalence of PCOS

**Fig. 2:**
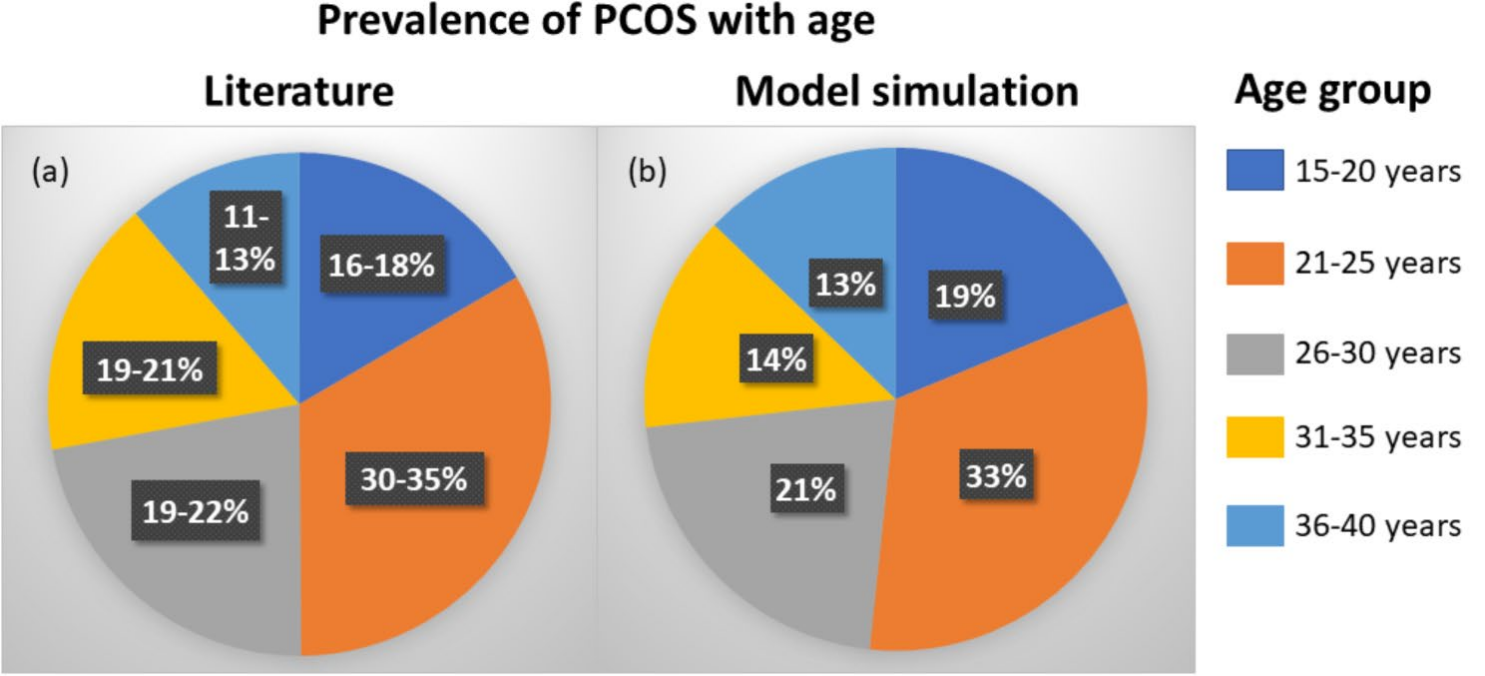
Prevalence of PCOS based on different age groups. Pie chart showing the prevalence from (**a**) published epidemiological data, (**b**) population generated in-silico for this study using model simulation. Same age groups are depicted in same colour

The simulated population was compared to the prevalence rate from the published data [45] (Fig. 2). The comparison results show similar prevalence of PCOS in different age groups. The age group of 15–20 years indicate prevalence rate of 16–18% and 19% in published data and simulated population respectively. For the age-group of 21–25 years, 26–30 years, 31–35 years and 36–40 years, the reported prevalence was 30–35%, 19–22%, 19–21% and 11–13% respectively whereas the simulated prevalence was 33%, 21%, 14% and 13% respectively.

### Effect of individual nutraceuticals on PCOS characteristics

The individual nutraceutical simulation results were analysed for their effects on five PCOS characteristics as mentioned in the PCOS scoring system. The effect of each nutraceutical has been measured in terms of fold change. Fold change was obtained by calculating ratio between levels of hormones or metabolites in healthy condition and levels of biochemical parameters in PCOS condition. The healthy condition is hence defined as a fold change of 1. All the eight nutraceuticals were analysed for individual effect on the PCOS condition. Compounds which could show effectiveness in treating at least one PCOS characteristic were deemed suitable to be included in the optimal combination for product formulation.

**Fig. 3:**
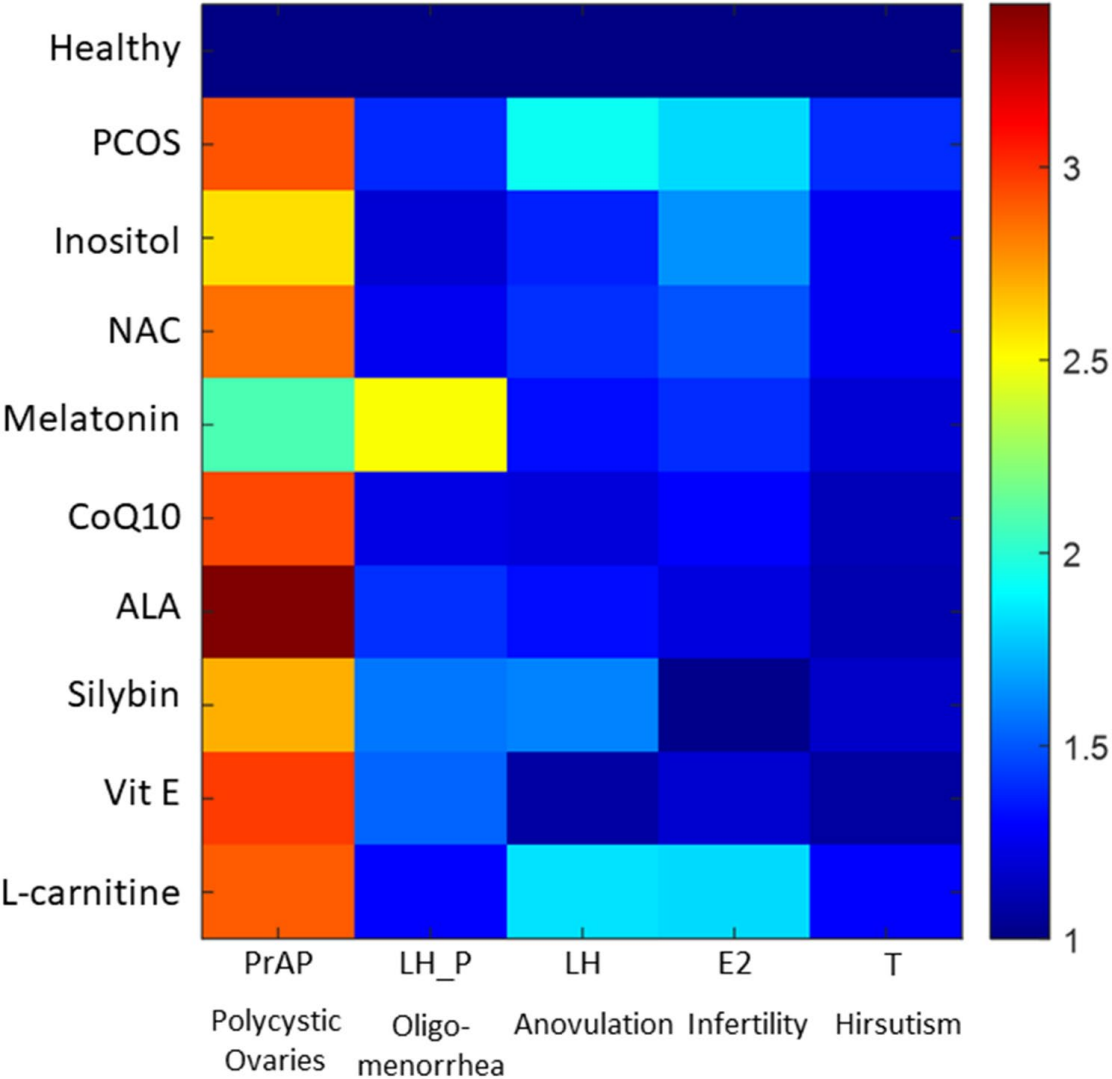
Heatmap comparing the effect of eight shortlisted nutraceuticals on PCOS characteristics. Expression levels (displayed as fold change from healthy condition) are represented by means of a colour scale in which blue and red represent healthy condition and PCOS respectively. PrAP: Pre-antral follicle mass, LH_P: Time between two LH surge

Figure 3 indicates that melatonin was most effective in treating PCOM amongst eight nutraceuticals by reducing the size of the pre-antral follicle. Inositol, NAC and CoQ10 were found to affect the anovulation by reducing the LH surge. Results show that melatonin, Vit E and CoQ10 showed reduced the cycle length thereby improving oligomenorrhea. ALA, silybin and Vit E were found to be effective in treating infertility by improving the levels of estradiol hormone. Results also suggests that nutraceuticals ALA, silybin and Vit E showed improvement in hirsutism by reducing testosterone hormone levels. Out of the eight shortlisted nutraceuticals, only L-carnitine was not able to show significant change in any PCOS characteristic. Therefore, the effective seven nutraceuticals were shortlisted for optimal nutraceutical combination analysis.

### Assessment of optimal nutraceutical combination

The shortlisted seven nutraceuticals were grouped into various combinations based on individual simulation results and the possible synergy between the shortlisted compounds which is known from the literature. These combinations were analysed to compare and finalize the six most potent nutraceutical combinations for treating PCOS.

Myo-inositol was included as a base in four out of six combinations [(i) Myo-inositol (2 g), Melatonin (2 mg), ALA (600 mg), (ii) Myo-inositol (2 g), Melatonin (2 mg), ALA (600 mg), N-acetyl cysteine (NAC) (600 mg), (iii) CoQ10 (300 mg), NAC (600 mg), Myo-inositol (2 g), (iv) CoQ10 (300 mg), ALA (600 mg), Myo-inositol (2 g)] as it showed the potential to treat four out of five PCOS characteristics considered in scoring system. The remaining two combinations had vitamin E as base [(i) Silybin (60 mg), Vitamin E (750 mg), CoQ10 (300 mg), (ii) Melatonin (2 mg), ALA (600 mg), Vitamin E (750 mg)], since vitamin E also showed significant improvement (< 0.0001) for oligomenorrhoea. Detailed analyses for all the combinations and their dosages are discussed in the supplementary file (Fig. S2 and Fig. S3). Our analysis revealed myo-inositol (2 g), melatonin (2 mg) and ALA (600 mg of R-lipoic acid) as the optimal nutraceutical combination for PCOS treatment.

**Fig. 4:**
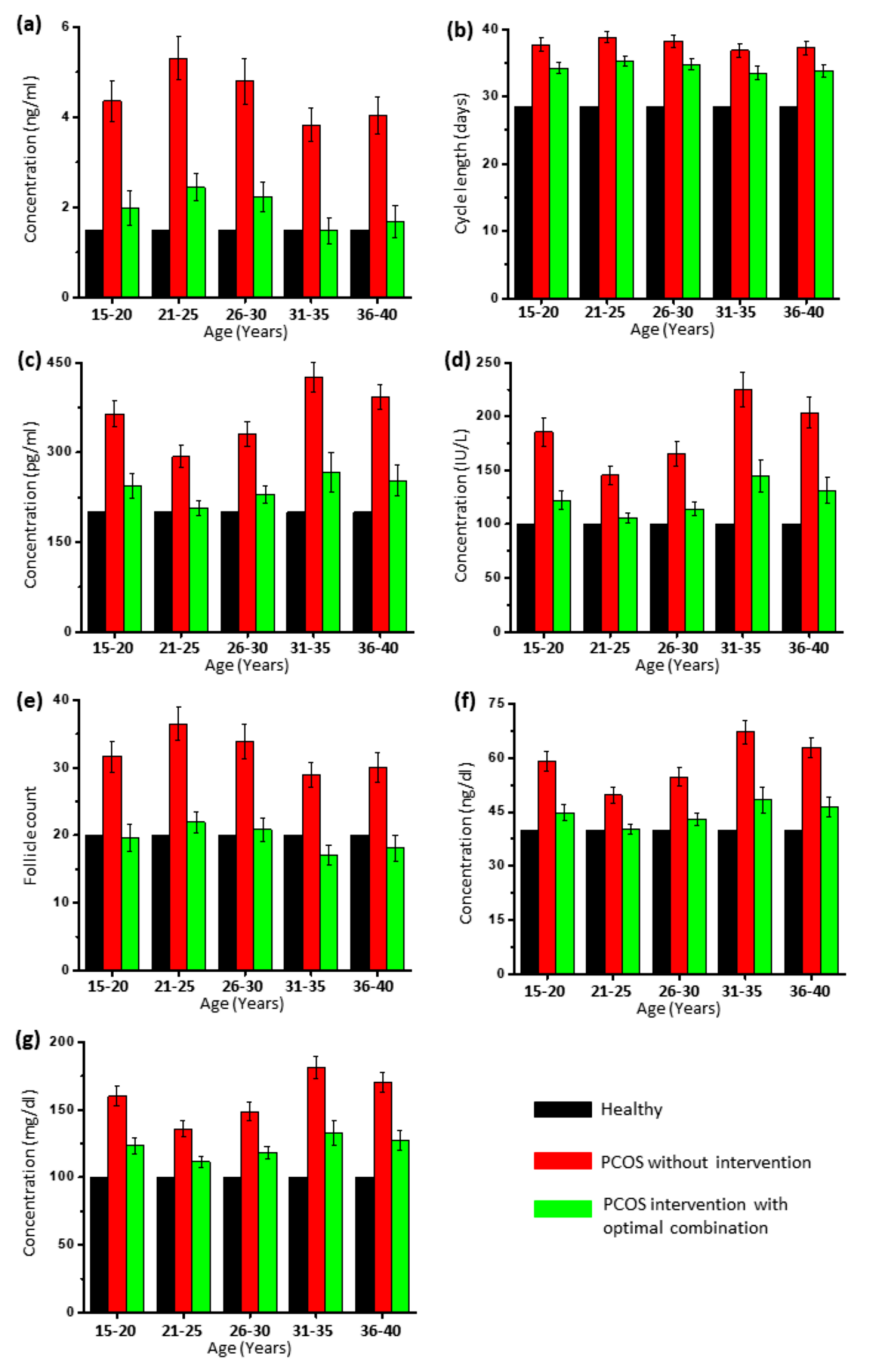
Age group-wise comparison of the effect of optimal nutraceutical combination on (**a**) serum anti-Müllerian hormone levels, (**b**) menstrual cycle length (Oligomenorrhea), (**c**) serum estradiol levels, (**d**) serum LH levels, (**e**) pre-antral follicle (PCOM), (**f**) serum testosterone levels, (**g**) serum triglycerides. T-test indicates *p* < 0.0001

The population analysis with the above-mentioned optimal nutraceutical combination interventions showed a significant (*p* < 0.0001) improvement in all PCOS characteristics mentioned in the PCOS scoring system. Figure 4 shows the categorization of the population into different age groups and the effect of interventions with the best combination. Non-significant differences in PCOS scores between ages suggest that the use of the best combination can benefit all age groups.

**Fig. 5:**
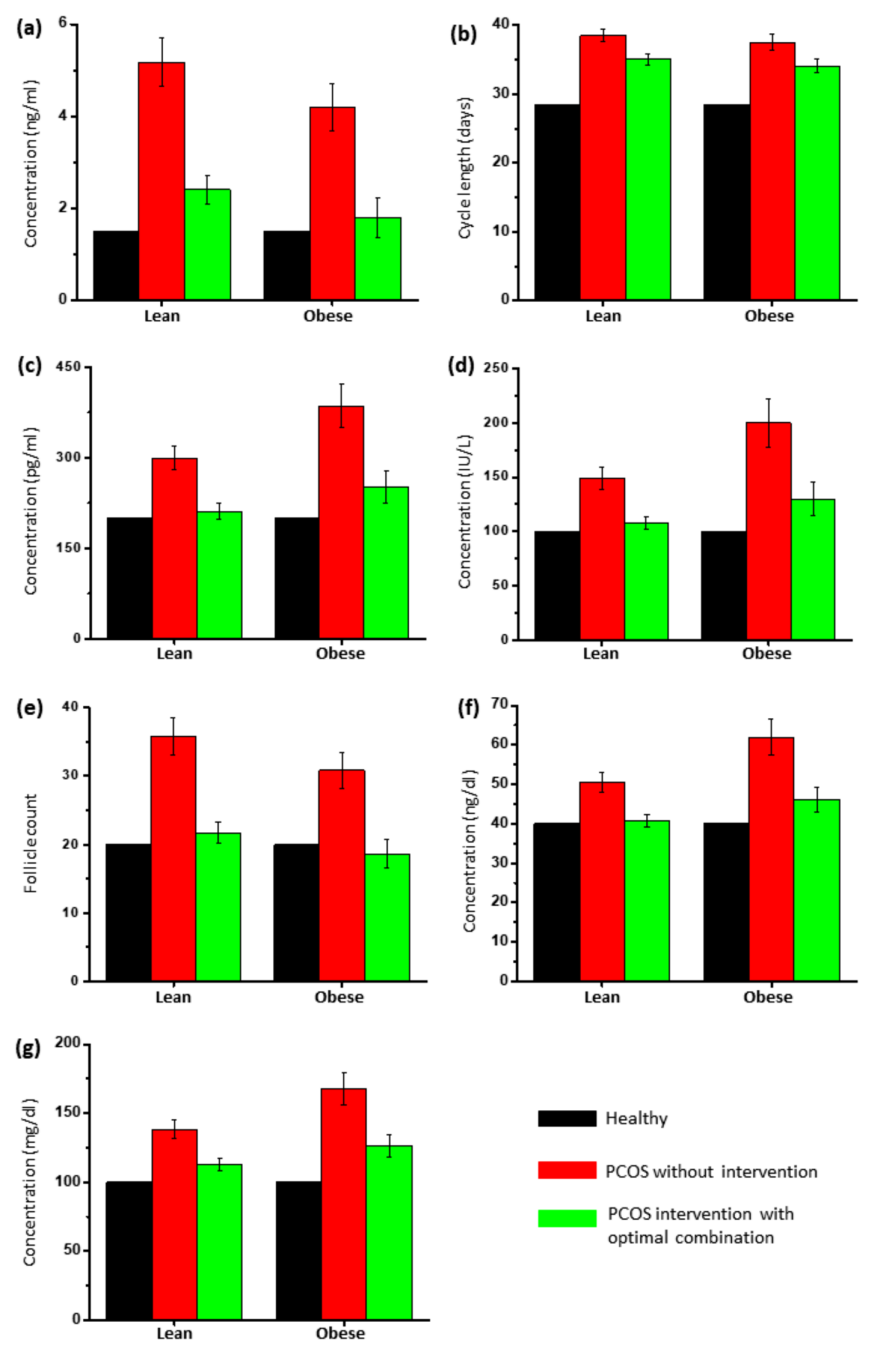
Effect of optimal nutraceutical combination on (**a**) serum anti-Müllerian hormone levels, (**b**) menstrual cycle length (Oligomenorrhea), (**c**) serum estradiol levels, (**d**) serum LH levels, (**e**) pre-antral follicle (PCOM), (**f**) serum testosterone levels, (**g**) serum triglycerides in lean and obese population. T-test indicates *p* < 0.0001

Population analysis was performed for lean and obese individuals with varying testosterone levels. The results shown in Fig. 5 depict that the intervention with optimal nutraceutical combination shows a significant reduction (*p* < 0.0001) in testosterone levels for both lean (40.76 ng/dl ± 1.62) and obese (46.08 ng/dl ± 3.13) PCOS populations. The intervention group also showed a reduction in LH and E2 levels. This implies that the best optimal combination may be effective for infertility treatment as these hormones are required for implantation. Tables 1 and 2 show the descriptive statistics for lean and obese PCOS with the best optimal combination.

**Table 1:** Statistical analysis showing the effect of optimal nutraceutical combination on PCOS phenotypes in the lean PCOS population

PCOS characteristics	Clinical parameters	Lean PCOS control group*	Lean PCOS intervention group*	*p*-value
Polycystic ovaries	Pre antral follicle (count)	35.82 (2.67)	21.75 (1.61)	< 0.0001
Anovulation	Cycle length (days)	38.54 (0.96)	35.04 (0.88)	< 0.0001
Oligomenorrhea	LH (IU/L)	148.97 (10.12)	107.73 (4.24)	< 0.0001
Infertility	Estradiol (pg/ml)	299.83 (20.03)	211.16 (13.86)	< 0.0001
Hirsutism	Testosterone (ng/dl)	50.61 (2.54)	40.76 (1.62)	< 0.0001
Infertility	AMH (ng/ml)	5.17 (0.52)	2.40 (0.31)	< 0.0001
Lean/Obese PCOS	Triglycerides (mg/dl)	138.33 (6.58)	112.83 (4.21)	< 0.0001

*Mean (SD)

**Table 2:** Statistical analysis showing the effect of optimal nutraceutical combination on PCOS phenotypes in the obese PCOS population

PCOS characteristics	Clinical parameters	Obese PCOS control group*	Obese PCOS intervention group*	*p*-value
Polycystic ovaries	Pre antral follicle (count)	30.80 (2.64)	18.64 (2.14)	< 0.0001
Anovulation	Cycle length (days)	37.52 (1.14)	34.10 (1.04)	< 0.0001
Oligomenorrhea	LH (IU/L)	199.73 (22.44)	129.87 (15.13)	< 0.0001
Infertility	Estradiol (pg/ml)	386.09 (35.10)	251.87 (27.12)	< 0.0001
Hirsutism	Testosterone (ng/dl)	61.96 (4.62)	46.08 (3.13)	< 0.0001
Infertility	AMH (ng/ml)	4.18 (0.52)	1.79 (0.42)	< 0.0001
Lean/Obese PCOS	Triglycerides (mg/dl)	167.71 (11.97)	126.59 (8.12)	< 0.0001

*Mean (SD)

## Discussion

This study presents an in-silico analysis of effects of various nutraceuticals in treating PCOS characteristics. The eight nutraceuticals shortlisted for simulation analysis were based on their maximum benefits and minimum side effects published in the literature. Model simulation analysis for individual nutraceuticals showed seven nutraceuticals effective in treating various PCOS characteristics. Myo-inositol alone showed improvement in all PCOS characteristics except PCOM. Clinical trials have observed folic acid combination with myo-inositol, but not folic acid alone, have resulted in better outcomes for the PCOM characteristic [46–49]. Also, administering the myo-inositol along with the isoforms of inositol such as D-Chiro-inositol (DCI) in the specific ratio of 40:1, respectively, has clinically demonstrated the improved ovulatory function [50].

The combination analysis results suggest inositol, melatonin, and ALA as the best optimal combination effective in maintaining a hormonal balance of LH, AMH, E2, and testosterone. Therefore, a formulation comprising the selected ingredients (myo-inositol (2 g), melatonin (2 mg) and ALA (600 mg)) can be beneficial in alleviating the spectrum of PCOS symptoms across the population. Previous studies have shown the effectiveness and synergy of inositol with melatonin [51] and alpha-lipoic acid [52] upon co-administration. The effects of three compounds revealed from the in-silico combination analysis were in line with their mechanisms of actions known from the literature [52, 53].

**Fig. 6:**
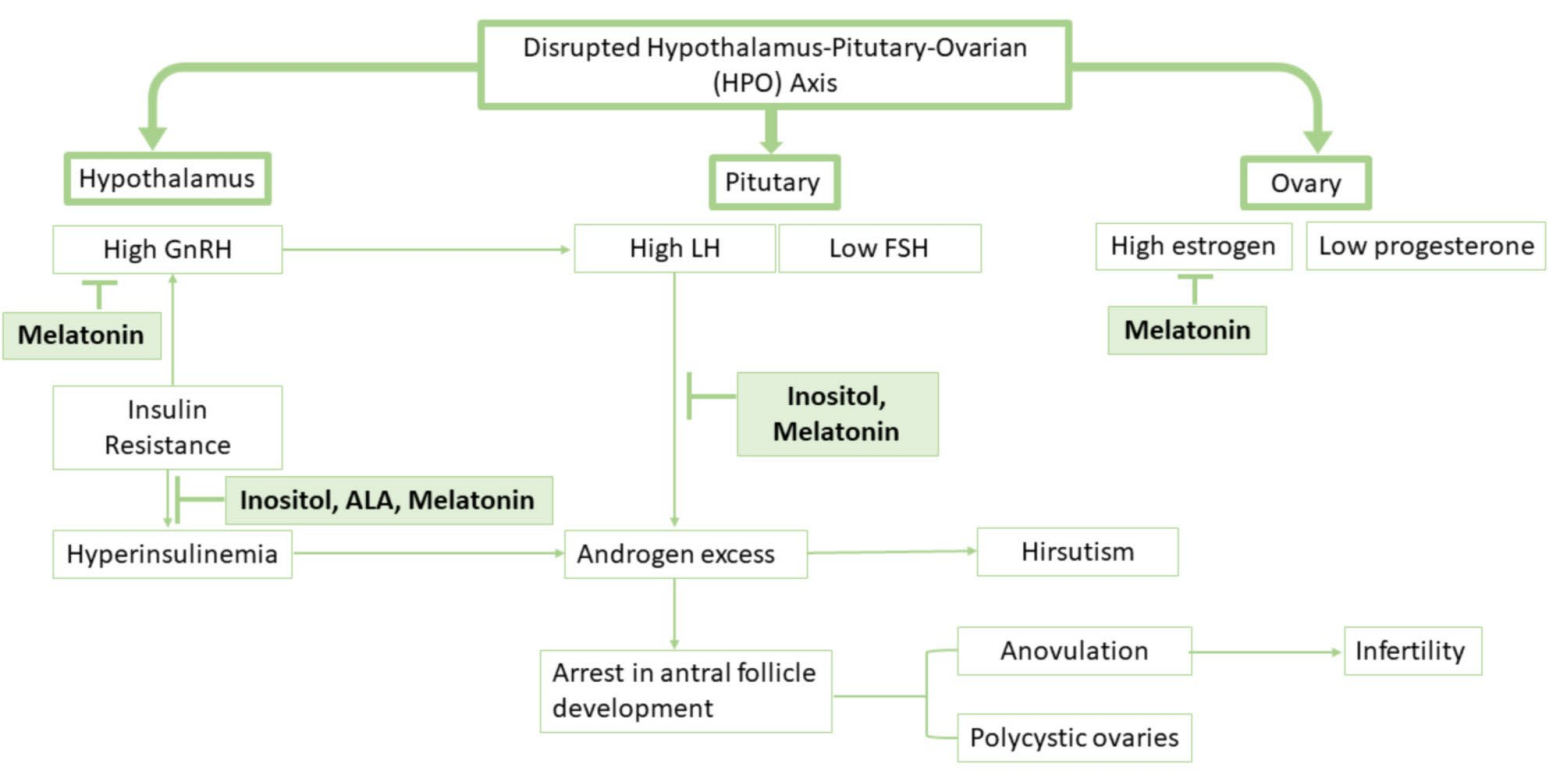
Detailed mechanism of action of the mapped ingredients for their benefits in PCOS. GnRH: Gonadotropin-releasing hormone, LH: Luteinizing hormone, FSH: Follicle-stimulating hormone, AMH: Anti-Müllerian hormone, ALA: Alpha-lipoic acid

Figure 6 mentions how the optimal nutraceutical combination has a role in alleviating the disrupted HPO-axis. Insulin resistance causes high pulsations of GnRH leading to increased LH activity [54]. High LH increases theca cell stimulation, which results in a hyperandrogenic milieu of the ovary. Hyperandrogenism arrests antral follicle development. Accumulation of pre-antral follicles causes polycystic ovaries that in turn contributes to elevated basal serum estrogen level [54]. Inositol, ALA, and melatonin are together known to reduce insulin resistance, key mechanism in PCOS pathogenesis [53, 55]. Melatonin restores the normal pulsation of GnRH, thereby indirectly normalizing LH levels. Myo-inositol and melatonin are known to normalize androgen levels, thereby inhibiting formation of polycystic ovarian morphologies [51]. Melatonin also has independently been involved in normalizing estrogen levels [53].

## Conclusion

In conclusion, the in-silico analysis of the optimal nutraceutical combination [(myo-inositol (2 g), melatonin (2 mg) and ALA (600 mg)] was observed to be a potential treatment for PCOS when supplemented for a duration of 6 months. The strength of our study is the validation of the nutraceutical effects on a large heterogeneous in-silico PCOS population. The heterogenous in-silico population has individuals from all reproductive age groups with lean and obese PCOS. Therefore, the efficacy of the optimal combination of nutraceuticals is predicted with higher confidence. One of the limitations of this study is that we assumed a population devoid of any deficiencies of vitamins and minerals. Low levels of micronutrients such as B-vitamins, vitamin D, chromium, magnesium, and selenium are found to have a crucial role in PCOS condition [56, 57]. Also, unlike clinical trials, modelling simulation cannot assess the physiological synergy of nutraceutical combinations. Future studies hence should focus on clinical validation of the effect and duration of administration for the obtained nutraceutical combination.

### Electronic supplementary material

Below is the link to the electronic supplementary material.

Supplementary Material 1

## Data Availability

All data generated or analysed during this study are included in this published article [and its supplementary information files].

## References

[1] Liu J, Wu Q, Hao Y (2021). Measuring the global disease burden of polycystic ovary syndrome in 194 countries: global burden of Disease Study 2017. Hum Reprod.

[2] Azziz R, Carmina E, Dewailly D (2006). Criteria for defining polycystic ovary syndrome as a predominantly hyperandrogenic syndrome: an androgen excess Society Guideline. J Clin Endocrinol Metab.

[3] Mihanfar A, Nouri M, Roshangar L, Khadem-Ansari MH, Polyphenols. Natural compounds with promising potential in treating polycystic ovary syndrome. Reprod Biol. 2021;21(2). 10.1016/j.repbio.2021.100500. 10.1016/j.repbio.2021.100500 33878526

[4] Reyes-Muñoz E, Sathyapalan T, Rossetti P (2018). Polycystic ovary syndrome: implication for drug metabolism on assisted Reproductive Techniques—A. Literature Rev Adv Therapy 2018.

[5] Mahran A (2016). The relationship between Anti-müllerian hormone and the clinical, biochemical and sonographic parameters in women with polycystic ovarian syndrome. Middle East Fertil Soc J.

[6] Chen W, Pang Y. Metabolic syndrome and PCOS: Pathogenesis and the role of metabolites. Metabolites. 2021;11(12). 10.3390/metabo11120869. 10.3390/metabo11120869 PMC8709086 34940628

[7] Azziz R, Carmina E, Chen Z (2016). Polycystic ovary syndrome. Nat Reviews Disease Primers 2016.

[8] Ganie M, Vasudevan V, Wani I, Baba M, Arif T, Rashid A (2019). Epidemiology, pathogenesis, genetics & management of polycystic ovary syndrome in India. Indian J Med Res.

[9] Mehreen TS, Ranjani H, Kamalesh R, Ram U, Anjana RM, Mohan V (2021). Prevalence of polycystic ovarian syndrome among adolescents and young women in India. J Diabetol.

[10] Bahadur A, Mundhra R, Kashibhatla J, Rajput R, Verma N, Kumawat M (2021). Prevalence of metabolic syndrome among women with different PCOS phenotypes - a prospective study. Gynecol Endocrinol.

[11] Tabassum F, Jyoti C, Sinha HH, Dhar K, Akhtar MS. Impact of polycystic ovary syndrome on quality of life of women in correlation to age, basal metabolic index, education and marriage. PLoS ONE. 2021;16(3). 10.1371/JOURNAL.PONE.0247486. 10.1371/journal.pone.0247486 PMC7946178 33690645

[12] Manique MES, Ferreira AMAP (2022). Polycystic ovary syndrome in adolescence: challenges in diagnosis and management. Revista Brasileira De Ginecol E Obstet.

[13] Nayaker BS, Thomas S, Ramachandran S, Loganathan S, Sundari M, Mala K (2017). Polycystic ovarian syndrome-associated cardiovascular complications: an overview of the association between the biochemical markers and potential strategies for their prevention and elimination. Diabetes Metabolic Syndrome: Clin Res Reviews.

[14] Lashen H (2010). Role of metformin in the management of polycystic ovary syndrome. Ther Adv Endocrinol Metab.

[15] Jensterle M, Kravos NA, Ferjan S, Goricar K, Dolzan V, Janez A (2020). Long-term efficacy of metformin in overweight-obese PCOS: longitudinal follow-up of retrospective cohort. Endocr Connect.

[16] Santini A, Cammarata SM, Capone G (2018). Nutraceuticals: opening the debate for a regulatory framework. Br J Clin Pharmacol.

[17] Gharaei R, Mahdavinezhad F, Samadian E (2021). Antioxidant supplementations ameliorate PCOS complications: a review of RCTs and insights into the underlying mechanisms. J Assist Reprod Genet.

[18] Zhang S, wei, Zhou J, Gober HJ, Leung WT, Wang L (2021). Effect and mechanism of berberine against polycystic ovary syndrome. Biomed Pharmacotherapy.

[19] Li MF, Zhou XM, Li XL. The Effect of Berberine on Polycystic Ovary Syndrome Patients with Insulin Resistance (PCOS-IR): A Meta-Analysis and Systematic Review. Evidence-based Complementary and Alternative Medicine. 2018;2018. 10.1155/2018/2532935. 10.1155/2018/2532935 PMC6261244 30538756

[20] Zilaee M, Mansoori A, Ahmad HS, Mohaghegh SM, Asadi M, Hormoznejad R (2020). The effects of soy isoflavones on total testosterone and follicle-stimulating hormone levels in women with polycystic ovary syndrome: a systematic review and meta-analysis. Eur J Contracept Reproductive Health Care.

[21] Poormoosavi SM, Behmanesh MA, Varzi HN, Mansouri S, Janati S (2021). The effect of follicular fluid selenium concentration on oocyte maturation in women with polycystic ovary syndrome undergoing in vitro fertilization/intracytoplasmic sperm injection: a cross-sectional study. Int J Reprod Biomed.

[22] Li Y, Tan Y, Xia G, Shuai J (2021). Effects of probiotics, prebiotics, and synbiotics on polycystic ovary syndrome: a systematic review and meta-analysis. Crit Rev Food Sci Nutr.

[23] Unfer V et al. A deeper assessment of ω3-poly-unsaturated fatty acids in polycystic ovary syndrome management. Comment on regidor. chronic inflammation in PCOS: The potential benefits of specialized pro-resolving lipid mediators (SPMs) in the improvement of the re. Int J Mol Sci. 2021;22(18):14–16. 10.3390/ijms221810114. 10.3390/ijms221810114 PMC8467655 34576277

[24] Mombaini E, Jafarirad S, Husain D, Haghighizadeh MH, Padfar P (2017). The impact of Green Tea supplementation on anthropometric indices and inflammatory cytokines in women with polycystic ovary syndrome. Phytother Res.

[25] Wei W, Zhao H, Wang A (2012). A clinical study on the short-term effect of berberine in comparison to metformin on the metabolic characteristics of women with polycystic ovary syndrome. Eur J Endocrinol.

[26] Giuliani C, Iezzi M, Ciolli L (2017). Resveratrol has anti-thyroid effects both in vitro and in vivo. Food Chem Toxicol.

[27] Kort DH, Lobo RA (2014). Preliminary evidence that cinnamon improves menstrual cyclicity in women with polycystic ovary syndrome: a randomized controlled trial. Am J Obstet Gynecol.

[28] Hajimonfarednejad M, Nimrouzi M, Heydari M, Zarshenas MM, Raee MJ, Jahromi BN (2018). Insulin resistance improvement by cinnamon powder in polycystic ovary syndrome: a randomized double-blind placebo controlled clinical trial. Phytother Res.

[29] Contreras-Bolívar V, García-Fontana B, García-Fontana C, Muñoz-Torres M (2021). Mechanisms involved in the relationship between vitamin D and insulin resistance: impact on clinical practice. Nutrients.

[30] Kaminska K, Grzesiak M (2021). The relationship between vitamin d3 and insulin in polycystic ovary syndrome-a critical review. J Physiol Pharmacol.

[31] Heshmati J, Omani-Samani R, Vesali S (2018). The effects of supplementation with chromium on insulin resistance indices in women with polycystic ovarian syndrome: a systematic review and Meta-analysis of Randomized clinical trials. Horm Metab Res.

[32] Sheikhhossein F, Amini MR, Shahinfar H, Djafari F, Safabakhsh M, Shab-Bidar S (2020). Effects of chromium supplementation on inflammatory biomarkers: a systematic review and dose-response meta-analysis of randomized controlled trials. Eur J Integr Med.

[33] Pourteymour Fard Tabrizi F, Hajizadeh-Sharafabad F, Vaezi M, Jafari-Vayghan H, Alizadeh M, Maleki V (2020). Quercetin and polycystic ovary syndrome, current evidence and future directions: a systematic review. J Ovarian Res.

[34] Sadeghi A, Djafarian K, Mohammadi H, Shab-Bidar S (2017). Effect of omega-3 fatty acids supplementation on insulin resistance in women with polycystic ovary syndrome: Meta-analysis of randomized controlled trials. Diabetes Metabolic Syndrome: Clin Res Reviews.

[35] Hendrix AO, Hughes CL, Selgrade JF (2014). Modeling endocrine control of the Pituitary–Ovarian Axis: androgenic influence and Chaotic dynamics. Bull Math Biol.

[36] Aversa A, La Vignera S, Rago R (2020). Fundamental concepts and novel aspects of polycystic ovarian syndrome: Expert consensus resolutions. Front Endocrinol (Lausanne).

[37] Maleki V, Jafari-Vayghan H, Kashani A (2019). Potential roles of carnitine in patients with polycystic ovary syndrome: a systematic review. Gynecol Endocrinol.

[38] Guo YM, Sun TC, Wang HP, Chen X (2021). Research progress of melatonin (MT) in improving ovarian function: a review of the current status. Aging.

[39] Devi N, Boya C, Chhabra M, Bansal D (2021). N-acetyl-cysteine as adjuvant therapy in female infertility: a systematic review and meta-analysis. J Basic Clin Physiol Pharmacol.

[40] Zhang J, Xing C, Zhao H, He B (2021). The effectiveness of coenzyme Q10, vitamin E, inositols, and vitamin D in improving the endocrine and metabolic profiles in women with polycystic ovary syndrome: a network Meta-analysis. Gynecol Endocrinol.

[41] Haghighatdoost F, Gholami A, Hariri M (2020). Alpha-lipoic acid effect on leptin and adiponectin concentrations: a systematic review and meta-analysis of randomized controlled trials. Eur J Clin Pharmacol.

[42] Reed BG, Carr BR. The normal menstrual cycle and the control of Ovulation. Endotext. Published Online August 5, 2018.

[43] Chahal S, Goyal P, Sodhi R (2021). Multiple molecular pathways unfolding the pathophysiology of polycystic ovary syndrome. Ann Rom Soc Cell Biol.

[44] Gaziano JM, Hennekens CH, O’Donnell CJ, Breslow JL, Buring JE (1997). Fasting triglycerides, High-Density Lipoprotein, and risk of myocardial infarction. Circulation.

[45] Purohit S, Rai S, Kalvit S, Purohit S, Rai S, Kalvit S (2021). Prevalence of polycystic ovarian syndrome and its association with circulatory gonadotropins (luteinizing hormone and follicle-stimulating hormone) and prolactin in different reproductive age groups: a brief survey. J Reproductive Healthc Med.

[46] Regidor PA, Schindler AE, Lesoine B, Druckman R. Management of women with PCOS using myo-inositol and folic acid. New clinical data and review of the literature. Horm Mol Biol Clin Investig. 2018;34(2). 10.1515/hmbci-2017-0067. 10.1515/hmbci-2017-0067 29498933

[47] Costantino D, Minozzi G, Minozzi F, Guaraldi C (2009). Metabolic and hormonal effects of myo-inositol in women with polycystic ovary syndrome: a double-blind trial. Eur Rev Med Pharmacol Sci.

[48] Papaleo E, Unfer V, Baillargeon JP et al. Myo-Inositol in patients with polycystic ovary syndrome: a novel method for ovulation induction. http://dx.doi.org/101080/09513590701672405. 2009;23(12):700–3. 10.1080/09513590701672405. 10.1080/09513590701672405 17952759

[49] Tabatabaie M, Amiri S, Golestan M, Azadeh J, Sene A, Zandieh Z. The effect of Myo Inositol supplement on molecular regulation of folliculogenesis, steroidogenesis, and assisted reproductive technique outcomes in patients with polycystic ovarian syndrome. Mol Biol Rep. 2021;0123456789. 10.1007/s11033-021-06833-9. 10.1007/s11033-021-06833-9 35040006

[50] Nordio M, Basciani S, Camajani E (2019). The 40:1 myo-inositol/D-chiro-inositol plasma ratio is able to restore ovulation in PCOS patients: comparison with other ratios. Eur Rev Med Pharmacol Sci.

[51] Russo M, Forte G, Oliva MM, Laganà AS, Unfer V. Melatonin and myo-inositol: supporting reproduction from the oocyte to birth. Int J Mol Sci. 2021;22(16). 10.3390/ijms22168433. 10.3390/ijms22168433 PMC8395120 34445135

[52] Genazzani AD (2020). Integrative treatment with inositols and lipoic acid for insulin resistance of PCOS. Gynecol Reproductive Endocrinol Metabolism.

[53] Mojaverrostami S, Asghari N, Sc M, Khamisabadi M, Khoei HH, Abbas A (2019). The role of melatonin in polycystic ovary syndrome: a review production and hosting by knowledge E. Int J Reprod Biomed.

[54] Liao B, Qiao J, Pang Y (2021). Central Regulation of PCOS: abnormal neuronal-Reproductive-Metabolic circuits in PCOS Pathophysiology. Front Endocrinol (Lausanne).

[55] Genazzani AD. Expert’s opinion: integrative treatment with inositols and lipoic acid for insulin resistance of PCOS– Gynecological and Reproductive Endocrinology & Metabolism. Published online 2020.

[56] Sharma P, Gupta V, Kumar K, Khetarpal P (2022). Assessment of serum elements concentration and polycystic ovary syndrome (PCOS): systematic review and Meta-analysis. Biol Trace Elem Res Published Online January.

[57] Günalan E, Yaba A, Yılmaz B (2018). The effect of nutrient supplementation in the management of polycystic ovary syndrome-associated metabolic dysfunctions: a critical review Elif. J Turk Ger Gynecol Assoc.

