# Supplementary material for: Efficacy of optimal nutraceutical combination in treating PCOS characteristics: an in-silico assessment
Source: BMC Endocr Disord. 2024 Mar 29;24:44. doi: 10.1186/s12902-024-01571-y (PMC10979615; doi:10.1186/s12902-024-01571-y)
Supplement: Supplementary file 1 — Supplementary Material 1 [file 12902_2024_1571_MOESM1_ESM.docx]

**Supplementary File**

Shortlisting of nutraceuticals: An extensive literature search was conducted across various platforms such as PubMed, EMBASE, the Cochrane Library and Google scholar. Scientific papers containing randomised controlled trials, systematic reviews, and meta-analysis for PCOS treatment through nutraceuticals were gathered. Data were extracted for the identified compounds, and their molecular functions were assessed. Certain nutraceutical compounds were filtered out due to insufficient data^1–6^ (Berberine, Rutin, Resveratrol, Catechins, Gallic acid, Quercetin and other bioflavonoids, Curcumin, Cinnamon, Soy, Vitamin A, Black cohosh (*Cimicifuga racemose*), Fenugreek seed extract, flaxseed, linseed, selenium, probiotics, prebiotics, symbiotic). Additional reasons of exclusion of certain compounds were their potential side-effects. Long-term resveratrol administration was associated with goitrogenic effects^7^. Omega 3 fatty acid, catechin and berberine supplementation observed gastro-intestinal symptoms^8–10^ while adverse events of cinnamon included headache, heartburn symptoms, menstrual cramps, and nausea with diarrhea^11^, rash and itchiness^12^. After analysing the shortlisted ingredients for their comparative benefits, few compounds were further eliminated from investigation due to uncertain efficacy^13–17^ (E.g., Vitamin D, chromium, quercetin) and non-reproducible results^8,18^ (E.g., omega-3 fatty acids). Finally, the analysis revealed the most efficient 8 nutraceutical ingredients which addressed most of the known symptoms and physiological perturbations of PCOS. Figure S1 shows a funnel approach to obtain the final list of shortlisted ingredients


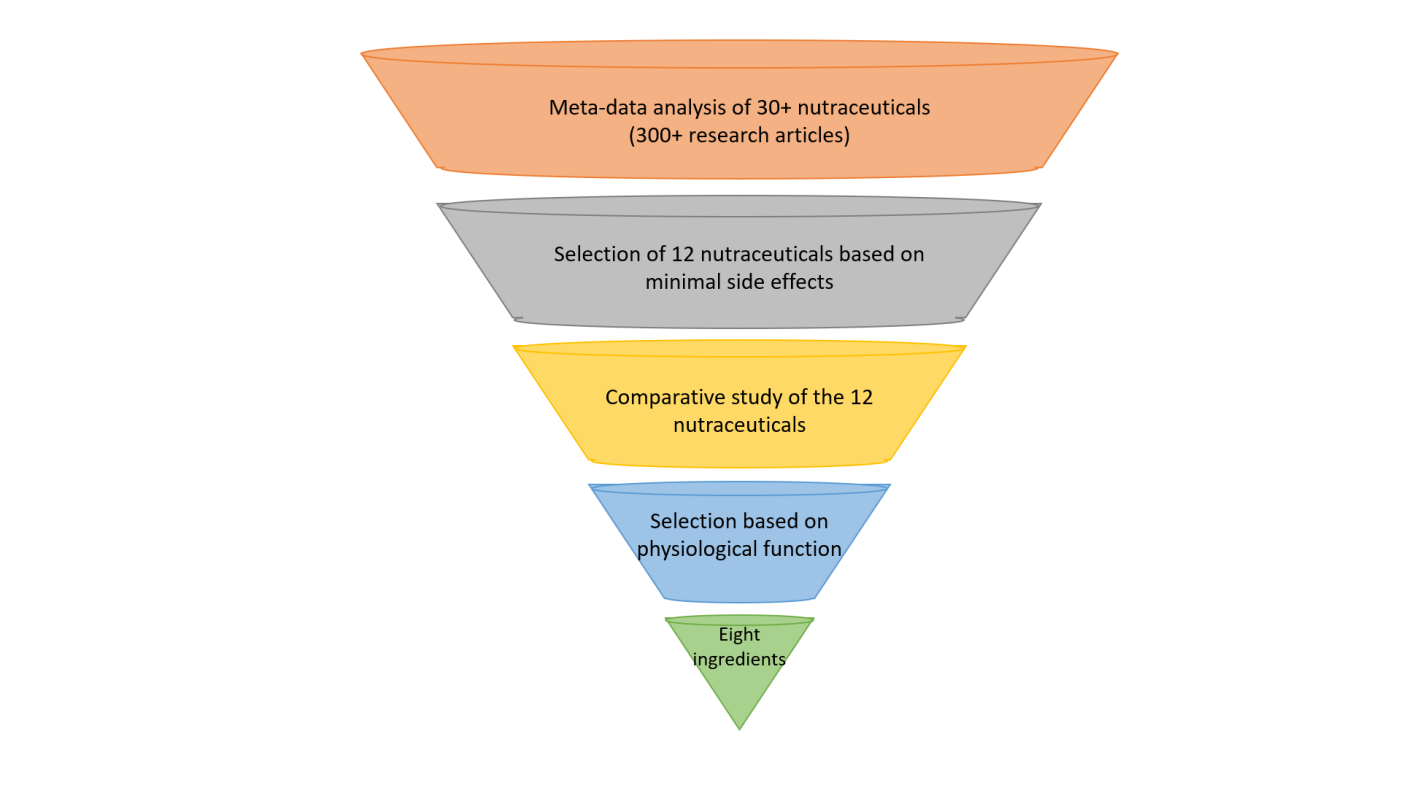


Figure S1: The approach used for shortlisting the active ingredients assessed on an in-silico PCOS population.

Optimal combination analysis: The pharmacokinetic profile of each nutraceutical was simulated for different doses using menstrual cycle model to study the physiological effect of nutraceuticals on PCOS characteristics and combined effect of nutraceuticals was assessed through optimal combination analysis. The further shortlisted 7 nutraceuticals were grouped into various combinations based on model simulation ranking results and possible compound synergy known from the literature before finalizing six most potential combinations. Myo-inositol was included in four combinations as it showed the potential to treat four out of five PCOS characteristics considered in scoring system. The remaining two combinations had Vitamin E as base for product formulation since vitamin E also showed significant improvement in the PCOS scores.

Myo-inositol based combinations:

1. Myo-inositol (2g), Melatonin (2mg), ALA (600mg)
2. Myo-inositol (2g), Melatonin (2mg), ALA (600mg), N-acetyl cysteine (NAC) (600mg)
3. CoQ10 (300mg), NAC (600mg), Myo-inositol (2g)
4. CoQ10 (300mg), ALA (600mg), Myo-inositol (2g)

Vitamin E based combinations:

1. Silybin (60mg), Vitamin E (750mg), CoQ10 (300mg)
2. Melatonin (4mg), ALA (600mg), Vitamin E (750mg)

Combination analysis results for formulations with vitamin E as a base is shown in Figure S2. It can be observed that both the combinations were not able to treat anovulation and oligomenorrhea. Furthermore, simulations performed with Myo-inositol as base (Figure S3), indicated that combination 1 [Myo-inositol (2g), Melatonin (2mg), ALA (600mg)] and combination 2 [Myo-inositol (2g), Melatonin (2mg), ALA (600mg), NAC (600mg)] shows similar results. Combination 3 [CoQ10 (300mg), NAC (600mg), Myo-inositol (2g)] and combination 4 [CoQ10 (300mg), ALA (600mg), Myo-inositol (2g)] had no effect on polycystic ovaries and anovulation.

Since NAC is known to effective only as an adjunct, among combination 1 and combination 2 of Myo-inositol-based combinations, we suggest Myo-inositol (2g), Melatonin (2mg) and ALA (600 mg of R-lipoic acid) as the optimal combination for a formulation.


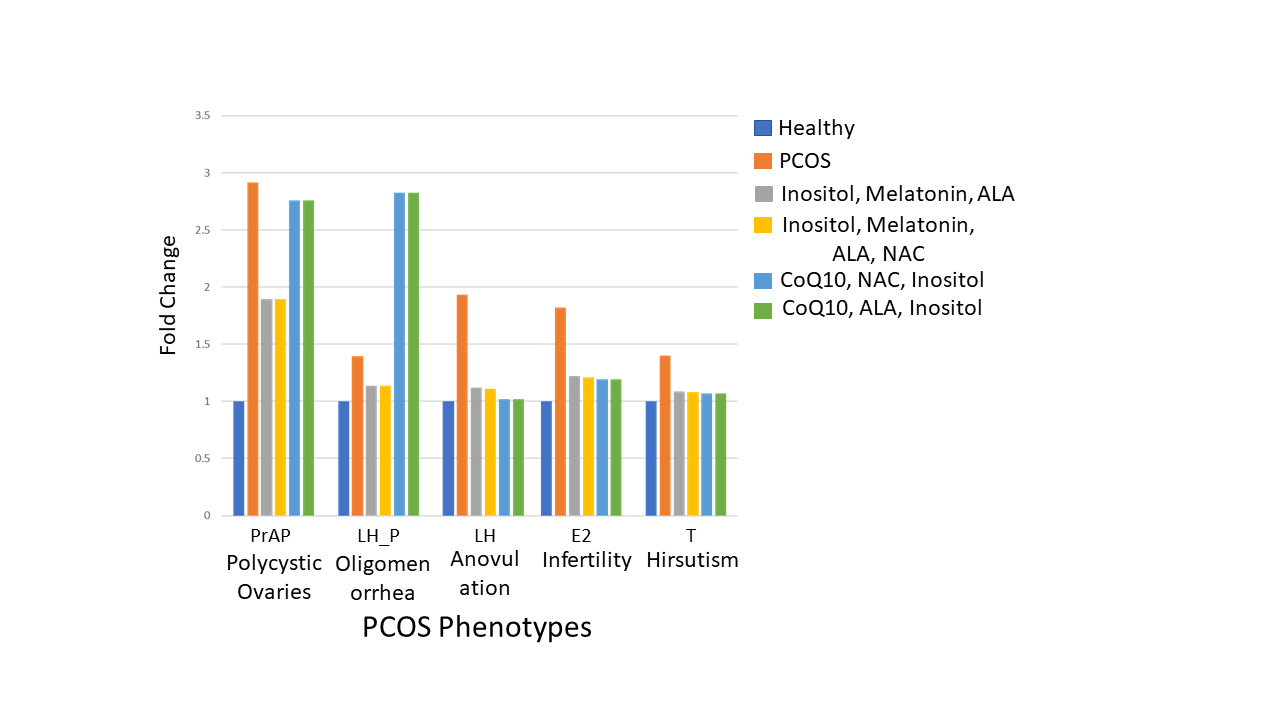


Figure S3: Combination analysis comparing the effect of combinations with and without melatonin for product formulation. For maximum effect the fold change should be minimum or close to 1 (healthy condition).


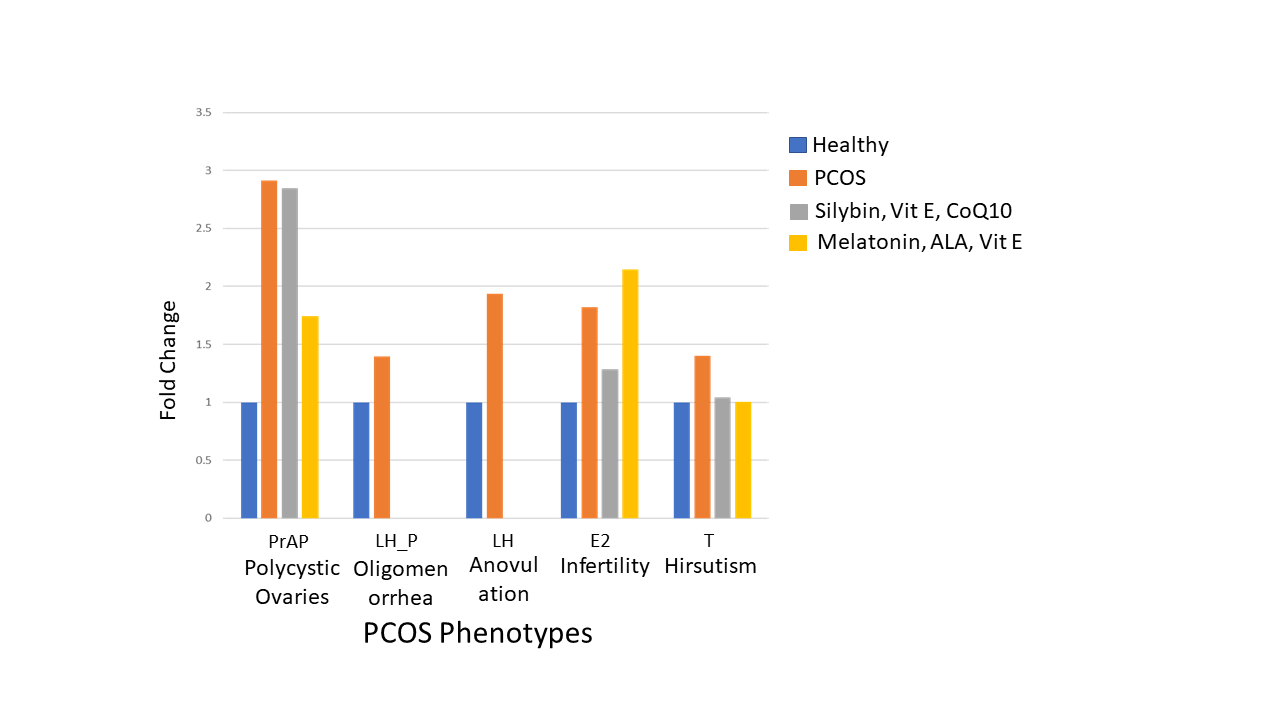


Figure S2: Combination analysis comparing the effect of different combinations with Vitamin E as base for product formulation. For maximum effect the fold change should be minimum or close to 1 (healthy condition).

Simulation results for dynamic analysis with optimal combination:

Simulations were performed in MATLAB using delayed differential equations for 7 menstrual cycles (210 days). The simulation compares the effect of intervention with and without optimal combination (Myo-inositol (2g), Melatonin (4 mg), ALA (600 mg)). The results show decrease in menstrual cycle length from 40 (PCOS without intervention) to 32 days (PCOS with intervention) (Figure S4). The effect of intervention also resulted in decrease in serum testosterone, pre antral follicles, anti-Müllerian hormone (AMH), triglycerides, LH, estradiol levels as well as reduced menstrual cycle length (Figure S5).


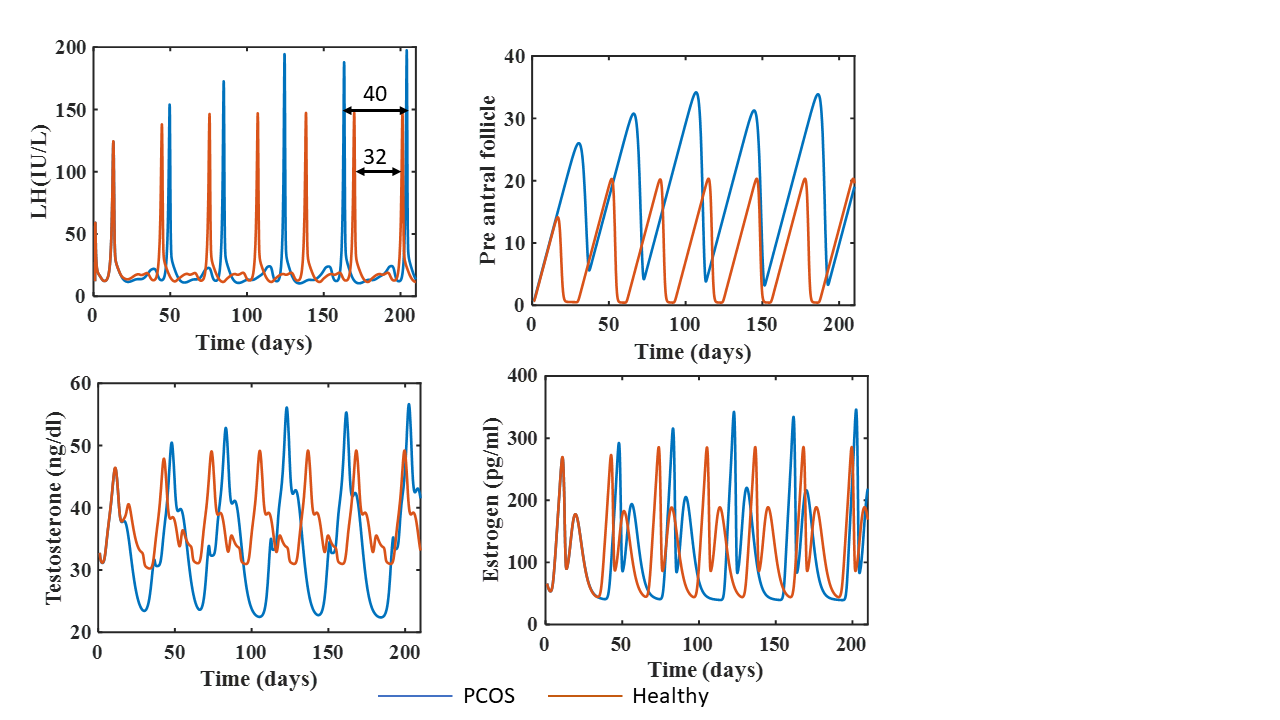


Figure S4: Dynamic simulation analysis showing the effect of optimal nutraceutical combination in PCOS.


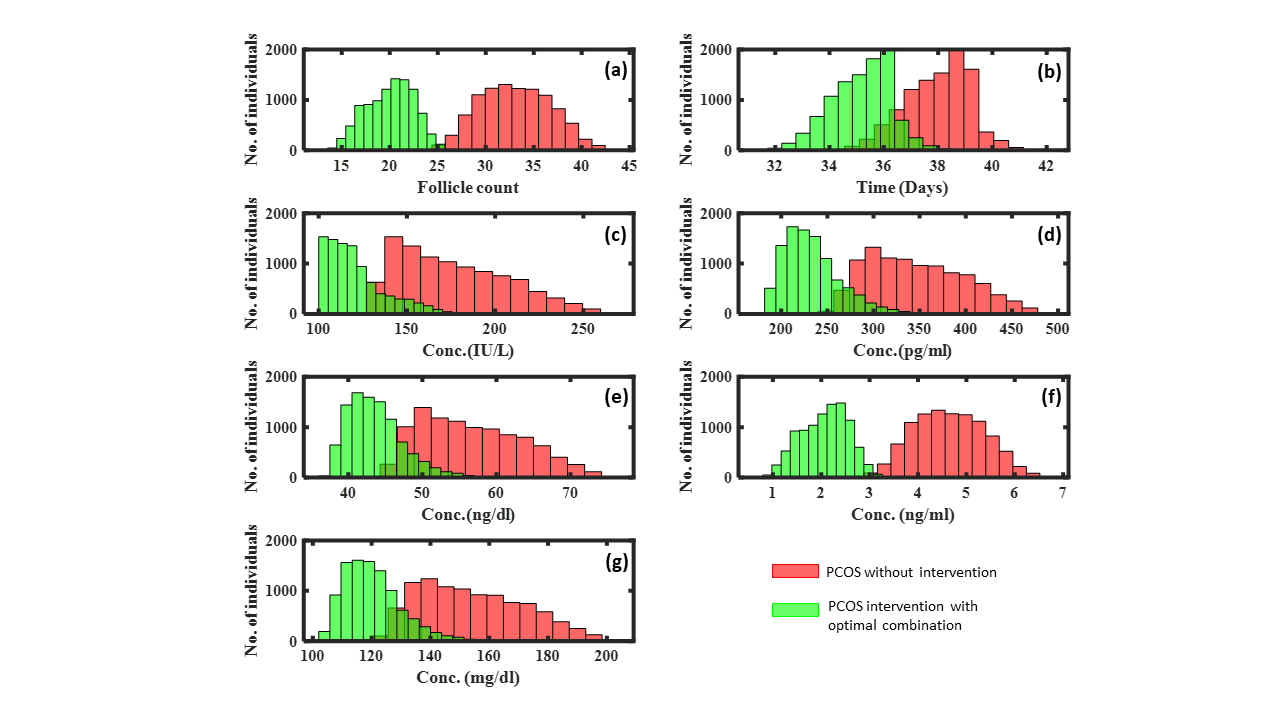


Figure S5: Population analysis showing the effect of intervention on (a) pre-antral follicle (PCOM), (b) menstrual cycle length (Oligomenorrhea), (c) serum LH levels, (d) serum estradiol levels, (e) serum testosterone levels, (f) serum anti-Müllerian hormone (AMH) levels, (g) serum triglyceride levels.

Table S1: Statistical analysis showing the effect of best optimal combination on PCOS phenotypes in PCOS population.

| **PCOS Phenotypes** | **Hormones** | **PCOS without intervention** | **PCOS with intervention** | **p-value** |
| --- | --- | --- | --- | --- |
| Polycystic ovaries | Pre antral follicle (count) | 33.11 ± 3.65 | 20.06 ± 2.45 | <0.0001 |
| Anovulation | LH surge time (days) | 37.99 ± 1.18 | 34.54 ± 1.07 | <0.0001 |
| Oligomenorrhea | LH (IU/L) | 176.35 ± 30.96 | 120.73 ± 16.29 | <0.0001 |
| Infertility | Estrogen (pg/ml) | 346.37 ± 51.94 | 233.12 ± 29.95 | <0.0001 |
| Hirsutism | Testosterone (ng/dl) | 56.73 ± 6.82 | 43.63 ± 3.68 | <0.0001 |
| Infertility | AMH (ng/ml) | 4.64 ± 0.72 | 2.07 ± 0.48 | <0.0001 |

Mean ± Standard deviation. T-test indicates p<0.0001.
